# Rapid Multiplexed Proteomic Screening for Primary Immunodeficiency Disorders From Dried Blood Spots

**DOI:** 10.3389/fimmu.2018.02756

**Published:** 2018-12-04

**Authors:** Christopher J. Collins, Irene J. Chang, Sunhee Jung, Remwilyn Dayuha, Jeffrey R. Whiteaker, Gesmar R. S. Segundo, Troy R. Torgerson, Hans D. Ochs, Amanda G. Paulovich, Si Houn Hahn

**Affiliations:** ^1^Seattle Children's Research Institute, Seattle, WA, United States; ^2^Division of Medical Genetics, Department of Medicine, University of Washington School of Medicine, Seattle, WA, United States; ^3^Fred Hutchinson Cancer Research Center, Seattle, WA, United States; ^4^Setor de Alergia e Imunologia Pediátrica, Ambulatório de Pediatria, Departamento de Pediatria, Universidade Federal de Uberlândia, Uberlândia, Brazil; ^5^Department of Pediatrics, University of Washington School of Medicine, Seattle, WA, United States

**Keywords:** peptide immunoaffinity enrichment coupled to SRM (immuno-SRM), Stable Isotope Standards and Capture by Anti-Peptide Antibodies (SISCAPA), Primary Immunodeficiency Disorders (PIDD), Wiskott-Aldrich Syndrome (WAS), X-linked Agammaglobulinemia (XLA), Severe Combined Immunodeficiency (SCID), newborn screening (NBS), Dried Blood Spot (DBS)

## Abstract

**Background:** Primary immunodeficiency disorders (PIDD) comprise a group of life-threatening congenital diseases characterized by absent or impaired immune responses. Despite the fact that effective, curative treatments are available with optimal clinical outcomes when diagnosed early, newborn screening does not exist for the majority of these diseases due to the lack of detectable, specific biomarkers or validated methods for population-based screening. Peptide immunoaffinity enrichment coupled with selected reaction monitoring mass spectrometry (immuno-SRM) is a sensitive proteomic assay, involving antibody-mediated peptide capture, that allows for concurrent quantification of multiple analytes. This assay has promise for use in potential newborn screening of PIDDs that lead to diminished or absent target proteins in the majority of cases.

**Objective:** To determine and evaluate if a multiplex assay based on immuno-SRM is able to reliably and precisely distinguish affected patients with X-linked agammaglobulinemia (XLA), Wiskott-Aldrich Syndrome (WAS), and CD3ϵ-associated severe combined immunodeficiency (SCID) from one another and from unaffected normal control dried blood spot (DBS) samples.

**Methods:** We performed a blinded, multiplexed analysis of proteolytically-generated peptides from WASp, BTK, and CD3ϵ (for WAS, XLA, and SCID, respectively) in DBS samples from 42 PIDD patients, 40 normal adult controls, and 62 normal newborns. The peptide ATPase copper transporting protein (ATP7B) 1056 was simultaneously monitored for quality assurance purposes.

**Results:** The immuno-SRM assays reliably quantified the target peptides in DBS and accurately distinguished affected patients from normal controls. Analysis of signature peptides found statistically significant reduction or absence of peptide levels in affected patients compared to control groups in each case (WASp and BTK: *p* = 0.0001, SCID: *p* = 0.05). Intra and inter-assay precision ranged from 11 to 22% and 11 to 43% respectively; linearity (1.39–2000 fmol peptide), and stability (≤ 0.09% difference in 72 h) showed high precision for the multiplexed assay. Inter-laboratory assay comparison showed high concordance for measured peptide concentrations, with R^2^ linearity ≥ 0.97 for the WASp 274, CD3ϵ 197, BTK 407, and ATP7B 1056 peptides.

**Conclusion:** Immuno-SRM-based quantification of proteotypic peptides from WASp, BTK, and CD3ϵ in DBS distinguishes relevant PIDD cases from one another and from controls, raising the possibility of employing this approach for large-scale multiplexed newborn screening of selective PIDDs.

## Introduction

Primary immunodeficiency disorders (PIDDs) are a collection of diverse congenital diseases characterized by aberrant or impaired immune responses. These include X-linked agammaglobulinemia (XLA, OMIM# 300755) caused by pathogenic variants in *BTK*, X-linked Wiskott-Aldrich Syndrome (WAS, OMIM# 301000) due to pathogenic variants in *WAS*, and Severe Combined Immunodeficiency (SCID), which can be caused by over 20 different genetic defects associated with T cell deficiency (1). Although genetically and clinically heterogeneous, these disorders lead to fatal infections unless treated early with intravenous immunoglobulin (IVIG), anti-microbials, or curative enzyme replacement, hematopoietic stem cell transplant (HSCT), or in some cases gene therapy (2–5). Early detection of PIDD can be lifesaving, but unfortunately, most affected infants are diagnosed only after developing devastating infections due to the lack of specific identifiable biomarkers or effective population-based screening methods. While T-cell receptor excision circle (TREC) analysis and kappa-deleting element recombination circle (KREC) screening from dried blood spots (DBS) on filter paper exist for SCID and some X-linked or autosomal recessive agammagobulinemias, respectively, newborn screening (NBS) methods for other PIDD do not exist (6–9).

Tandem mass spectrometry (MS/MS) was first applied to NBS in the 1990s, paving the way for rapid screening of multiple metabolites and thus several diseases from DBS samples collected at birth (10–15). Selected reaction monitoring mass spectrometry (SRM-MS) performed on triple quadrupole mass spectrometers further enabled the precise, high-throughput, and analytically-robust quantification of specific biomarkers; as such, it is now the standard of care in clinical newborn screening laboratories across the world (10, 16, 17).

MS/MS relies on the measurement of concentrated upstream metabolites for the detection of inborn errors of metabolism with specific enzyme deficiencies (11). This excludes its application to diseases such as PIDD, where no accumulated metabolites are present or currently verified. For this reason, protein-based assays such as flow cytometry or western blotting have been used as first-line investigative methods for diseases such as WAS and its milder phenotype, X-linked thrombocytopenia (XLT), where most pathogenic variants lead to absent or decreased protein products (18, 19). These approaches require that intact blood samples or isolated peripheral blood mononuclear cells (PBMC) from patients be available, making population-based screening or testing of patients from resource-poor areas impossible.

SRM-MS utilizes proteolytically-generated signature peptides as stoichiometric surrogates of the protein of interest. This may, in turn, be used to estimate the number of a particular cell-type expressing that protein in a sample (i.e., quantification of CD3ϵ for an indication of the amount of CD3+ T-cells in blood).The high specificity of MS for each signature peptide is conferred by three physiochemical properties—its mass, retention times upon high-performance liquid chromatography (HPLC) separation, and resultant target-specific fragmentation patterns (20). Recently, LC-MS/MS has recently been used to screen for Adenosine Deaminase (ADA) deficient SCID (21). Despite these advances, with a typical limit of quantification ranging from 100 to 1,000 ng protein/mL, the use of complex matrices such as blood or plasma often precludes accurate quantification of extremely low-abundance targets by SRM-MS based assays. This limits its applicability to many PIDD including XLA, SCID, and WAS that result in absent or decreased levels of target proteins expressed only intracellularly (22).

Peptide immunoaffinity enrichment coupled to SRM (immuno-SRM), also referred to as Stable Isotope Standards and Capture by Anti-Peptide Antibodies (SISCAPA), increases the sensitivity of SRM-MS assays by utilizing anti-peptide antibodies to purify and enrich peptides of interest from a complex biologic sample prior to SRM-MS analysis (23–27). This additional peptide affinity step, coupled to SRM-MS, lowers the limit of detection to the low pg protein/mL range from 1 mL of plasma that is suitable for the accurate quantification of very low abundance proteins in complex matrices such as DBS (24, 28–30).

We have previously demonstrated the ability of liquid chromatography (LC)-MS/MS to detect signature peptides from CD3ϵ, WASp, and BTK in proteolytically digested human PBMC lysates. WASp and BTK were chosen because mutations in these proteins are the source of WAS and XLA, respectively. In contrast, CD3ϵ was chosen in an attempt to develop a universal marker for the genetically heterogenous SCID. We hypothesized that the T-Cell lymphopenia exhibited by SCID patients would lead to a demonstrable decrease in CD3ϵ, itself a T-Cell marker. One or two surrogate signature peptides were chosen for each target protein based on factors including amenability to MS detection, uniqueness in the proteome, and absence of common single nucleotide polymorphisms. A decrease in protein concentration brought on by pathogenic mutations would therefore be reflected in a reduction in the measured concentration of signature peptides.

In a blinded study, peptide levels were quantified in normal control PMBCs but nearly undetectable in a disease-specific fashion in affected patients (31). We then applied the same proteomic method to show elevated levels of α-aminoadipic semialdehyde antiquitin and piperideine-6-carboxylate in DBS of patients with pyridoxine-dependent seizures, revealing the possibility of its application to NBS (32). To improve the sensitivity and reproducibility of the assay, we harnessed the immuno-SRM platform to quantify very low abundance peptides in DBS such as surrogate peptides of the ATP7B protein from patients with Wilson disease (WD). Our results demonstrated immuno-SRM's capability to detect ATP7B peptides in the low picomolar (pmol) range and reproducibly differentiating between patients with WD from unaffected controls (33). The goal of our present study is to expand this immuno-SRM method to the multiplexed analysis of a large cohort of patients with SCID, WAS, or XLA. Blinded analysis of patients and normal controls will test the potential utility of immuno-SRM for high-throughput and multiplexed, population-based newborn screening.

## Materials and Methods

### Patient Samples

This protocol was approved by the institutional review board of Seattle Children's Hospital. All subjects gave written informed consent in accordance with the Declaration of Helsinki. PIDD and normal control blood samples were obtained from the Seattle Children's Immunology Diagnostic Laboratory. Newborn DBS collected prior to March 2015 were retrieved from the Washington State Newborn Screening Laboratory (Shoreline, WA) after IRB approval. XLA DBS were collected from 20 suspected Vietnamese patients and shipped per regular mail to Seattle Children's Hospital. Genotypes of these patients by Sanger sequencing was previously reported (34). In total, DBS samples from 42 PIDD patients and 40 normal controls were obtained. Normal control and PIDD patient DBS were prepared by pipetting 70 μL of blood/12 mm spot onto filter paper cards (Protein Saver 903 Card, Whatman, Piscayaway,NJ), allowed to dry at room temperature overnight, and stored in sealed plastic bags at −80°C until use. Affected patient samples were shipped at room temperature from collection locations and stored at −80°C until use.

### Immuno-SRM Assay Reagents

ProteaseMAX Surfactant (no. V2072) and proteomics grade trypsin (no. V5113) were purchased from Promega (Madison, WI). Bovine serum albumin standard (200 mg/mL), and (3-[3-cholamidopropyl = dimethylammonio]-1-propanesulfonate) (CHAPS, no. PI28300) detergent were obtained from Fisher Scientific. Ammonium bicarbonate (40867-50G-F) was purchased from Fluka Analytical. Acetonitrile (no. A955), water (no. W6, LCMS optima grade), formic acid (no. PI28905), and phosphate-buffered saline (PBS, no. 10010-023) were obtained from Thermo Fisher Scientific (Waltham, MA).

Heavy stable isotope-labeled peptides were obtained from Anaspec (Fremont, CA). The stable isotope-labeled peptides were purified >95% by HPLC and the C-terminal arginine or lysine was labeled with [13C and 15N] atoms, resulting in a mass shift of +8 or +10 Da, respectively. Aliquots were stored in 5% acetonitrile/0.1% formic acid at −20°C until use.

Antibodies were immobilized on 2.8 μm Dynabeads Protein G magnetic beads (no. 10004D, Invitrogen, Carlsbad, CA) in a 1 μg antibody-to-2.5 μL of beads ratio. In brief, 250 μL of the beads were added to 1.5 mL Eppendorf tubes (022363204 Eppendorf) and washed twice with 250 μL of 1 × PBS + 0.03% CHAPS, followed by the addition of 100 μg of antibody and 1 × PBS + 0.03% CHAPS (no. 28300, Thermo Scientific) to yield a total 250 μL of volume. The antibodies were allowed to couple to the beads overnight with tumbling at 4°C. The next day, the antibodies were immobilized onto the beads with chemical cross-linking. Briefly, antibody beads were collected using magnetic pulldown, excess PBS was discarded, and 300 μL of freshly prepared 20 mM DMP (dimethyl pimelimidate dihydrochloride, no. D8388, Sigma) in 200 mM triethanolamine, pH 8.5 (no. T58300, Sigma) was added. The samples were tumbled for 30 min at room temperature, and the DMP in triethanolamine was discarded. Two-Hundred and Fifty microliter of 150 mM monoethanolamine (no. 411000, Sigma) was added and the beads were tumbled at room temperature for 30 min. The antibody beads were washed twice using 250 μL of 5% acetic acid + 0.03% CHAPS (5 min of tumbling at room temperate each time), and washed once more using 250 μL of 1 × PBS + 0.03% CHAPS. The ATP7B, BTK, WASp, and CD3ϵ antibody-linked beads were then washed and incubated in 5% acetic acid + 3% acetonitrile (ACN), washed with 250 uL of 1xPBS + 0.03% CHAPS, and the latter two steps were repeated once. All antibody-linked beads were washed with 250 μL of 1 × PBS + 0.03% CHAPS until neutral pH (7.0) was achieved, then resuspended in 250 μL of 1 × PBS + 0.03% CHAPS and 2.5 uL of NaN_3_ (52002-5G Sigma Aldrich) for anti-fungal properties and stored at 4°C until use.

### DBS Protein Extraction and Trypsin Digestion

All protein extraction and trypsin digestion steps were performed at Seattle Children's Research Institute (SCRI). For each sample (blinded normal controls or patients), one entire DBS spot (13 mm) containing ~70 μL blood was perforated into approximately 17 punches at 3-mm diameter with a standard leather punch tool. Final sample representation was WAS: *n* = 11, XLA: *n* = 26, SCID: *n* = 3 and normal controls (*n* = 40). The punches were placed in a 1.5 mL eppendorf tube, and 490 μL of 0.1% ProteaseMax in 50 mM ammonium biocarbonate (pH 8) was added into each tube. The tubes were vortexed for 1 h on the Eppendorf MixMate (Eppendorf, Hamburg, Germany), after which 10 μL of each sample were aliquoted and diluted 200-fold for Bradford assay to determine protein concentration. Disulfide bond reduction was performed with 2 M DTT at 5 mM, and an additional 490 μL of 0.1% ProteaseMax in 50 mM ammonium biocarbonate (pH 8) was added into each tube before incubation in 37°C water bath for ~30 min. Trypsin was then added at a 1:50 enzyme to protein ratio (w/w), and acetonitrile was added to a final concentration of 15%. The mixture was incubated in a 37°C water bath overnight for digestion before centrifugation for 10 min at 13,000 RPM before each supernatant was transferred to a new tube and dried in the Savant™ SpeedVac™ High Capacity Concentrator (Thermo Fisher Scientific (Waltham, MA). All trypsinized DBS digests were stored at −80°C until use.

For samples analyzed from the Washington State NBS laboratory, Five or Six 3-mm punches were used for protein extraction and digestion (*n* = 62). Procedures were identical to those for previous samples except that volumes were reduced as follows: 150 μL of 0.1% ProteaseMax and 0.78 μL DTT for each addition.

### Liquid Chromatography–Tandem Mass Spectrometry

Enriched samples were analyzed at two sites (one at SCRI and one at Fred Hutchinson Cancer Research Center), to examine the inter-laboratory variability in data acquisition, utilizing two separate LC-MS/MS systems and instrument configurations (described below). Measured peptide concentrations were then compared for method validation. Peptide parent and daughter ion spectra have been previously reported (31).

*At Seattle Children's Research Institute:* Instruments included a Waters Xevo TQ-XS MS with ionkey source technology connected to Waters M-Class Gradient and Loading pumps (Waters, Milford MA). Chromatographic solvents were A: H_2_O + 0.1% Formic Acid (FA) and B: ACN + 0.1% FA. Initially, peptides mixtures were loaded onto a M-Class Trap Symmetry 300 μm × 50 mm C18 column (100 Å, 5 μm) utilizing a constant flow of 98:2 A:B at 20 uL/min for 3 min. Subsequently, the flow was reversed and peptides were separated using gradient flow across a 150 μm × 100 mm BEH C18 ikey (130 Å, 1.7 μm). Gradient method programming is shown in Table 1. The peptides monitored in this location were BTK 407, WASp 289, WASp 274, ATP7B 1056, and CD3ϵ 197.

**Table 1:** LC method setup for signature peptide separation at Seattle Children's Research Institute and Fred Hutchinson Cancer Research Center.

**SCRI**		**FHCRC**	
**Time (min)**	**% B**	**Time (min)**	**% B**
0	5	0	1
1	5	4	1
11	45	24	40
13	85	25	90
15	85	26	90
17	5	27	1
20	5	35	1

*A = H_2_O + 0.1% Formic Acid, B = B: ACN + 0.1% Formic Acid*.

Parameters for transitions and collision energy (CE) were taken from a linear regression of previously optimized values in Skyline and those generated using Waters intellistart technology to identify the most intense fragments upon ionization. SRM transitions were acquired at unit/unit resolution in both the Q1 and Q3 quadrupoles with 5 ms dwell time and 3 ms pause between mass ranges, resulting in a cycle time of 1.5 s. All samples were run in a blinded fashion.

*Fred Hutchinson Cancer Research Center:* LC-MS was conducted on a SCIEX 5500 QTRAP mass spectrometer interfaced with an Eksigent 425 LC and Nanoflex Chip system. Chromatographic solvents were A: H_2_O + 0.1% Formic Acid (FA) and B: 90% ACN + 0.1% FA. Peptides were loaded on a 0.2 × 0.5 mm trap column (Reprosil-Pur AQ C18, 3 um, 120 A) at 2% B using a flow rate of 4 uL/min for 4 min. Peptides were eluted on a 0.075 × 150 mm column (Reprosil-Pur AQ C18, 3 um, 120 A) at 300 nL/min. The gradient program is shown in Table 1.

Collision energy settings were taken from Skyline (35). Transitions were acquired at unit/unit resolution with a 10 ms dwell time and 5 ms pause between mass ranges resulting in a cycle time of 0.75 s. All data were acquired in a blinded fashion.

### Data Analysis

All SRM data were analyzed and plotted using Skyline (MacCoss Lab Software, open source, Seattle, WA, https://skyline.ms/project/home/begin.view) (35). Endogenous target peptide concentrations were quantified by comparing the ratio of the peak area of the signature peptide to its IS added at a known concentration (100 fmol). Statistics were generated using Graphpad Prism (San Diego, CA). Receiver operating characteristic (ROC) curves were constructed using Graphpad Prism and a 95% confidence interval.

### Selection of Surrogate Peptides and Antibody Production

Surrogate peptides for BTK, WASp, and CD3ϵ were selected by *in silico* trypsin digestion and NCBI BLAST tools. Final peptide selections were made according to accepted major criteria for immuno-SRM development including peptide length, lack of post-transcriptional modifications, and uniqueness in the human genome by BLAST searching as previously described (31, 36, 37). Peptide selection and monoclonal antibody production for ATP7B signature peptides have been previously reported (33). Crude peptides were then screened empirically to determine suitability for detection and quantification by LC-MS/MS.

Affinity-purified rabbit polyclonal antibodies (pAb) were successfully generated against five peptides by Pacific Immunology (Ramona, CA). Briefly, signature peptides were synthesized with an N-terminal cysteine, conjugated to keyhole limpet hemocyanin (KHL) for immunization. Two New Zealand white rabbits were injected per peptide. pAbs for all selected peptides successfully underwent affinity-purification from 25 mL of antiserum. A monoclonal antibody was used for ATP7B peptide capture as described (38).

### Peptide Immunoaffinity Enrichment

Peptide immunoaffinity enrichment was performed at SCRI. DBS digests were resuspended in 1 × PBS + 0.03% CHAPS to yield a 1 μg/μL nominal protein digest concentration. Cross-linked, antibody-coated beads were added to a total mass of 2 μg pAb for each target along with 20 μL of 1M Tris pH 8.0 (15568-025 UltraPure Invitrogen). Isotope-labeled peptides were added as internal standards (IS). This suspension was incubated overnight with tumbling at 4°C to achieve peptide capture. The next day, the antibody bead: peptide complexes were washed twice with 100 μL PBS + 0.01% CHAPS and once in 100 μL 0.01% PBS + 0.01% CHAPS. Finally, the peptides were eluted by incubation in 30 μL of 5% acetic acid/3% ACN. Released peptides were stored at −80°C until analysis. For samples analyzed from the WA State NBS laboratory, procedures were identical to those for previous samples except that volumes were reduced as follows: 58.1 μL of 1x PBS + 0.03% CHAPS, 0.59 μg pAb for each peptide, 3.13 μL IS, and 12.5 μL TRIS.

### Method Performance Assessment

A response curve was performed to determine assay linearity and sensitivity in a background matrix of DBS. Punches from normal control DBS (4 punches per sample) were extracted using extraction buffer (ProteaseMax, ammonium bicarbonate) in triplicate. Trypsin digestion was performed on the extracted protein, and the digests were pooled to create a common background matrix. Heavy stable isotope standards were spiked into the digest and serially diluted to create samples with varying peptide amounts (2000, 200, 12.5, 4.17, 1.39, 0.69 fmol). Samples were then processed as described above.

Repeatability and intra- and inter-assay precision were characterized by performing measurement of endogenous (light) peptide signal over 5 separate days. Each sample was analyzed in 5 complete process replicates (including punches, extraction, digestion, enrichment, and mass spectrometry) per day.

Stability was assessed by comparing the endogenous peptide detected in DBS stored at room temperature for 1 day and 3 days to peptide detected in DBS preserved at −80°C in a sealed container. Each sample was processed as described above in triplicate. Percent difference was calculated at each time point.

### Inter-laboratory Validation of the Analytical Assay

DBS extractions, trypsin digestion, and peptide captures for patient samples were all performed exclusively at Seattle Children's Research Institute (SCRI). Peptide solutions eluted from antibody-beads were split into two 15 μl aliquots for analysis at SCRI and Fred Hutchinson Cancer Research Center (FHCRC) for inter-laboratory comparison of the analytical performance of the assay.

## Results

### Peptide Selection and Antibody Development

Selected peptide sequences, molecular weights, parent, and daughter ions are listed in Table 2.

**Table 2:** Protein targets and sequences used for immuno-SRM study.

**Disease**	**Protein**	**Peptide**	**Sequence**	**Mass**	**Parent ion (m/z)**	**Daughter ions (m/z)**
Severe combined immunodeficiency	CD3ϵ	CD3ϵ 197–205	DLYSGLNQR	1066.54	533.27 ++	674.36 (y6), 587.33 (y5), 530.30 (y4)
X-Linked Agammaglobulinemia	BTK	BTK 407–417	ELGTGQFGVVK	1135.62	567.81 ++	892.49 (y9), 835.46 (y8), 734.42 (y7), 677.40 (y6)
		BTK 545–558	YVLDDEYTSSVGSK	1563.72	781.86 ++	1187.51 (y11), 1072.48 (y10), 957.45 (y9), 828.41 (y8)
Wiskott-Aldrich syndrome	WASp	WASp 274–288	AGISEAQLTDAETSK	1521.76	760.88 ++	1192.57(y11), 1063.53 (y10), 992.49 (y9), 864.43 (y8), 751.35 (y7)
		WASp 289–304	LIYDFIEDQGGLEAVR	1838.94	919.47 ++	1186.61(y11), 1073.52 (y10), 944.48 (y9)

*Total mass, parent ion mass, and daughter ion information are also shown. ++ indicates doubly charged parent ion*.

Fragmentation patterns for the peptides of interest have been previously reported (31). Affinity-purified polyclonal antibodies (Pacific Immunology, Ramona, CA) were generated against all five peptides and pursued for use in human samples because of their ability to successfully capture their target sequences and the absence of background signals brought on by copurified peptide contaminants (26, 28, 39).

### Method Performance Assessment

**Figure 1:**
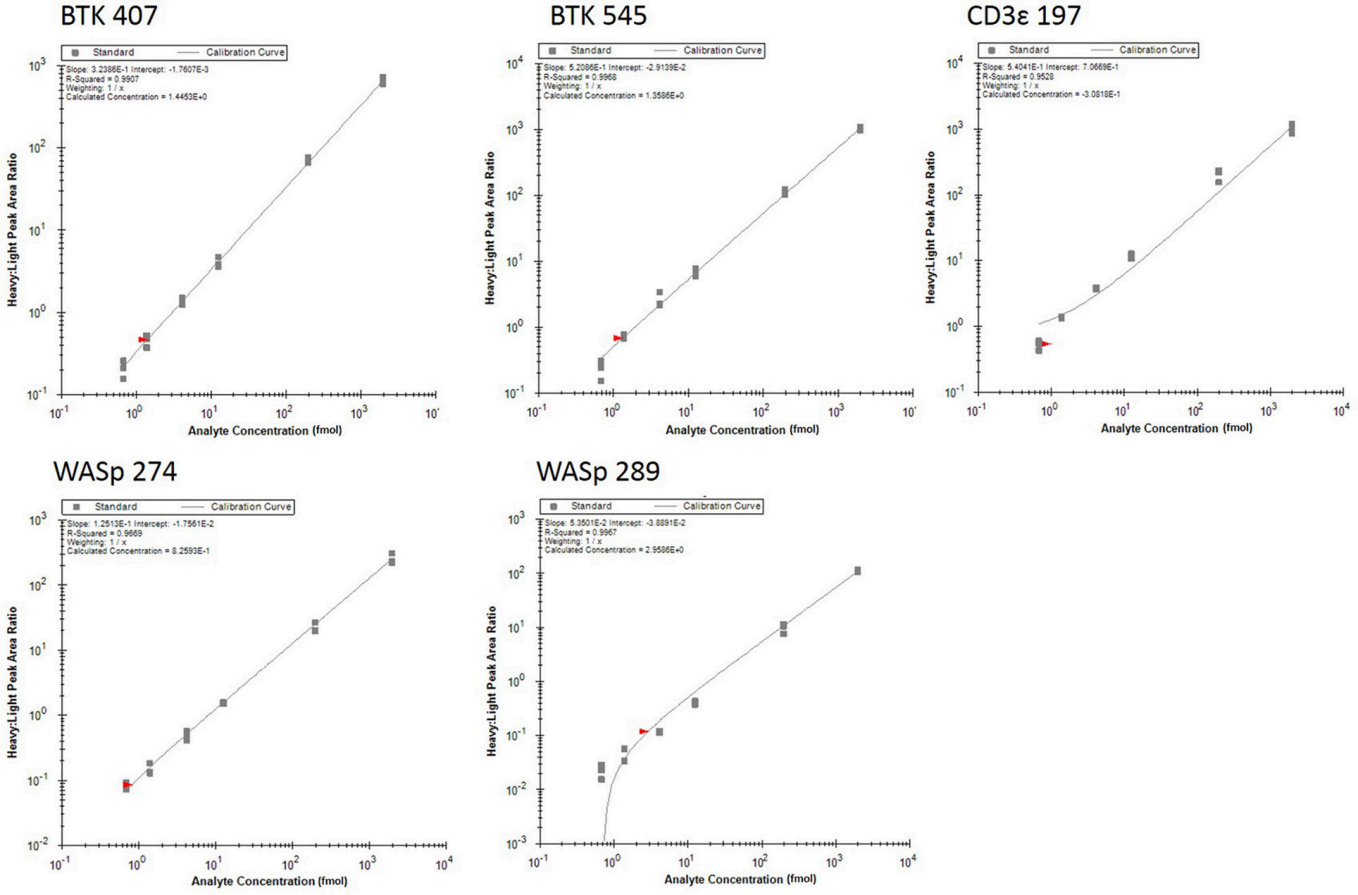
Response curves for peptides measured by the multiplexed immuno-SRM assay. Response curves plot the heavy: light peak area ratio as a function of heavy peptide concentration, measured in a background matrix of digested protein extracted from dried blood spots. The curves allow determination of the linear range and sensitivity of the assay. Each data point is plotted as a gray box and linear regression is plotted as a line. Regression fit parameters are reported in the corner of each plot.

Analytical figures of merit are reported in Table 3. Overall, the linear responses spanned a range from 1.39 to 2000 fmol of peptide (Figure 1).

**Table 3:** Analytical performance of signature peptides.

**Protein**	**Peptide**	**LLOD**	**ULOD**	**LLOQ**	**Median CV**	**Intra-assay CV**	**Inter-assay CV**	**Relative**	**Difference**
		**(fmol)**			**%**			**24 h**	**72 h**
CD3ϵ	CD3ϵ 197–205	0.69	2000	0.69	13	12	11	0.17	0.09
BTK	BTK 407–417	0.69	2000	1.39	10	14	25	0.32	−0.05
	BTK 545–558	0.69	2000	1.39	12	22	43	0.61	0.06
WASp	WASp 274–288	0.69	2000	0.69	17	11	12	−0.04	0.01
	WASp 289–304	1.39	2000	4.17	7	13	17	−0.19	0.03

The median coefficient of variation (CV) for all points on the response curve was 11%. Lower limits of quantification (LLOQ) were defined by the lowest point to yield a CV < 20%. LLOQs ranged from 0.69 to 12.5 fmol. There were five peptides detected above LLOQ in the DBS samples. Across all peptides, the mean intra-assay (i.e., within-day) variability ranged from 11 to 22% while the inter-assay (i.e., between-day) variability ranged from 11 to 43%. Of note, a single peptide (BTK 545-558) showed variability greater than 20% CV.

**Figure 2:**
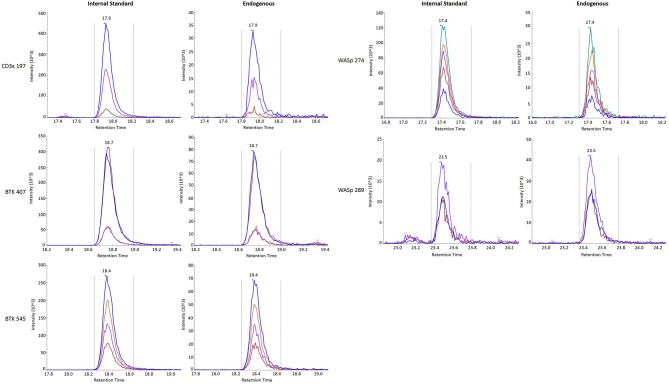
SRM traces for internal standard **(Left)** and endogenous **(Right)** signature peptides.

Finally, stability was assessed by comparing the endogenous (light) peptide detected in DBS stored at room temperature for 1 day and 3 days to peptide detected in DBS preserved at −80°C in a sealed container. Results are reported in Table 3. All five peptides had endogenous signal above the LLOQ and little variability over time. Representative SRM chromatograms for each peptide are shown in Figure 2.

**Figure 3:**
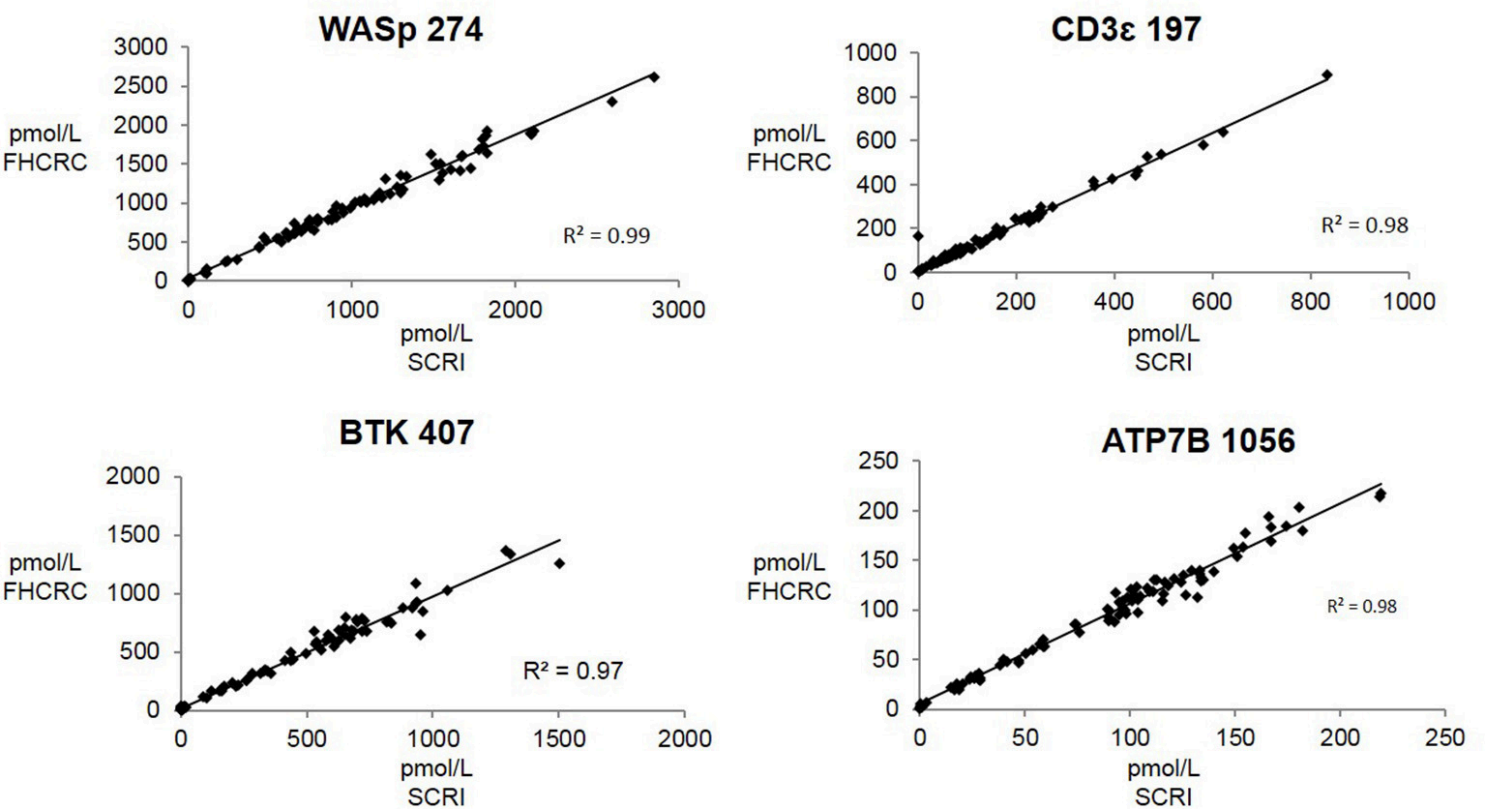
Inter-laboratory correlation in measured PIDD peptide concentrations. SCRI, Seattle Children's Research Institute; FHCRC, Fred Hutchinson Cancer Research Center.

**Figure 4:**
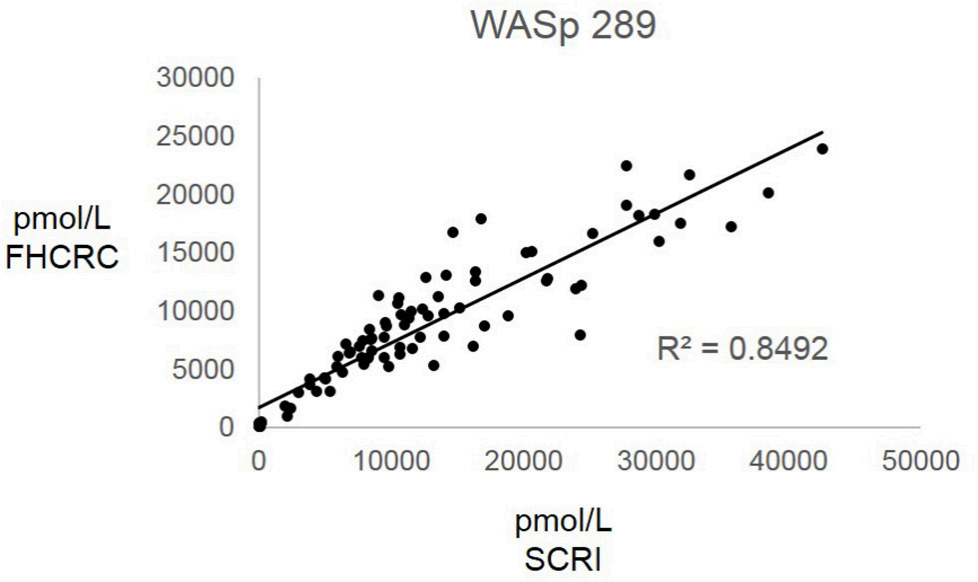
Inter-laboratory analytical validation of WASp 289. SCRI, Seattle Children's Research Institute; FHCRC, Fred Hutchinson Cancer Research Center.

Overall, there was high level of agreement between the concentrations quantified from samples prepared at one site and analyzed by two separate instrumental analyses. Correlation plots comparing the two measurements, Figure 3, show the linearity of measured concentrations with *R*^2^ values ≥ 0.97 in the cases of primary peptides WASp 274, CD3ϵ 197, BTK 407, and ATP7B 1056. The measurements of WASp 289 were found to correlate with *R*^2^ = 0.85 and would therefore be used as a secondary marker (Figure 4).

### Peptide Concentrations

**Figure 5:**
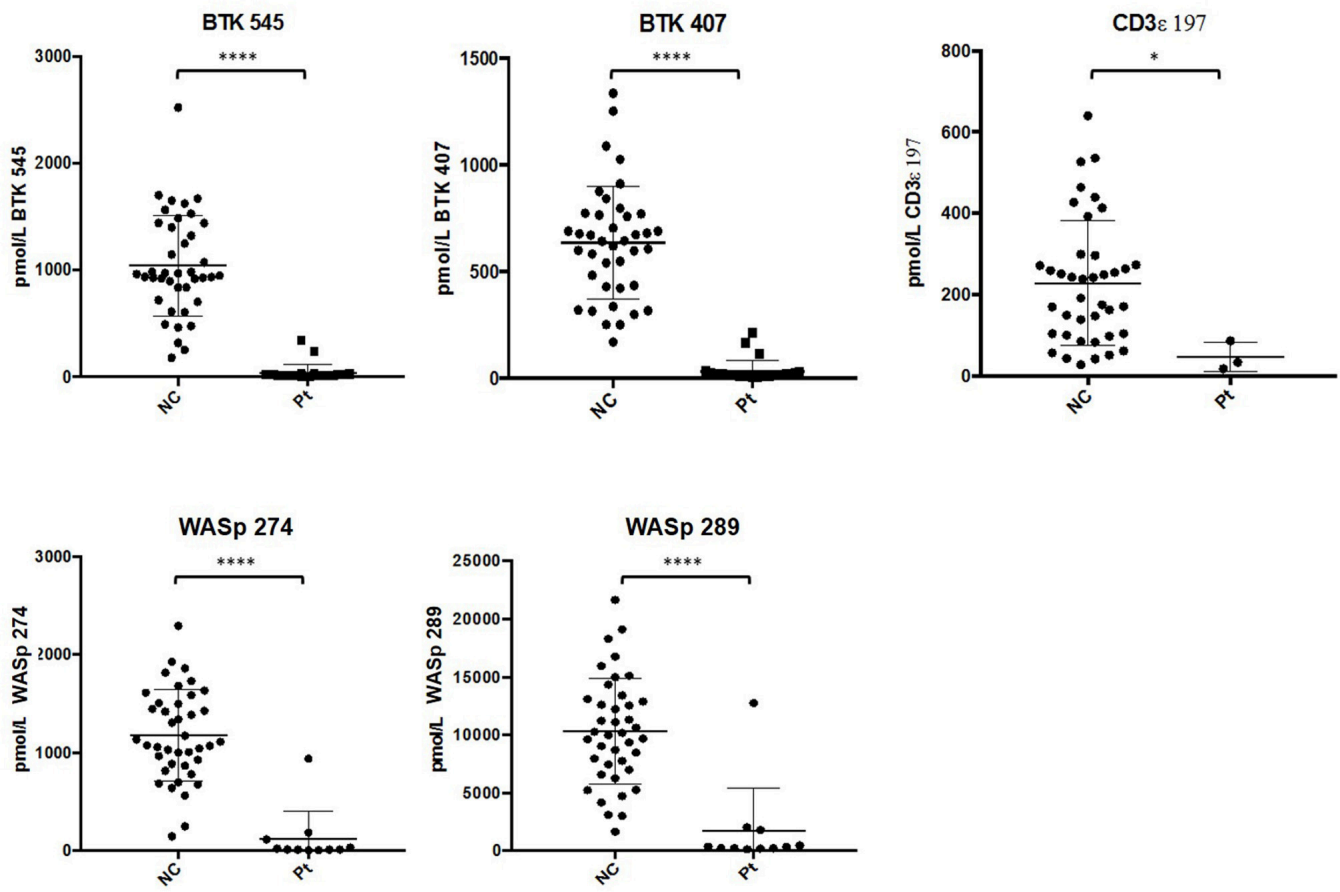
Differences in signature peptide levels between patients (WAS: *n* = 11, XLA: *n* = 26, SCID: *n* = 3) and normal controls (*n* = 40). Error bars indicate mean ± SD. WAS patient sample with high levels of WASp peptides was identified to be collected after bone marrow transplantation. ****p < 0.0001, *p < 0.05.

After analysis, normal controls were unblinded to define normal ranges for affected patient comparison. The average peptide concentrations from normal controls were as follows (average ± SD): BTK 545 = 1038.44 ± 465.77 pmol/L, BTK 407 = 635.09 ± 260.40 pmol/L, WASp 289 = 10326.98 ± 4513.13 pmol/L, WASp 274 = 1176.96 ± 456.68 pmol/L, and CD3ϵ = 228.68 ± 150.98 pmol/L. Analysis of signature peptides found statistically significant (*p* < 0.05–0.0001) reductions in patient peptide levels relative to control groups in each case (Figure 5).

Peptide levels in the majority of affected patients were significantly diminished or absent (Table 4). For each patient, the concentration of ATP7B 1056 was also determined using previously developed immuno-SRM methodology (33). These protein concentrations served as quality control (QC) measurements and their consistency across samples was used to assess digestion and process reproducibility (Table 5).

**Table 4:** Concentrations of signature peptides in a blinded patient cohort study.

**Patient**	**BTK 545 (pmol/L)**	**BTK 407(pmol/L)**	**WASp 274 (pmol/L)**	**WASp 289(pmol/L)**	**CD3ϵ 197 (pmol/L)**	**Immuno-SRMdiagnosis**	**Clinical diagnosis**	**Genotype**	**Notes**
1	2221.71	1362.07	2609.43	27763.21	246.07	Normal	X-linked CGD	*CYBB* Mutation	
2	1148.86	744.21	1028.71	11880.21	231.00	Normal	X-linked CGD	*CYBB* Mutation	
3	3.79	13.79	1919.71	23893.21	86.29	BTK	BTK	*BTK* c.1587_1589delA (p.N530Tfs26^*^)	Brother of #4
4	11.79	13.93	1623.07	17947.86	200.43	BTK	BTK	*BTK* c.1587_1589delA (p.N530Tfs26^*^)	Brother of #3
5	26.96	16.96	1280.39	17413.25	99.11	BTK	BTK	*BTK* c.1940T>C (p.L647P)	
6	20.21	11.36	257.00	4310.21	177.50	BTK	BTK	*BTK* c.763C>T (p.R255^*^)	
7	19.64	12.43	1121.29	18177.57	71.57	BTK	BTK	*BTK* c.1940T>C (p.L647P)	
8	23.36	23.82	637.00	8699.25	103.86	BTK	BTK	*BTK* c.1889T>A (p.M630K)	
9	21.71	13.36	495.00	6009.64	63.50	BTK	BTK	*BTK* c.1908+2delTAAGTGCTT	
10	237.64	113.14	548.00	7162.29	65.79	Normal	BTK	No mutation identified	
11	10.86	10.93	781.79	9599.00	81.71	BTK	BTK	*BTK* c.1768A>T (p.I590F)	
12	13.79	11.36	618.50	7024.36	65.64	BTK	BTK	No mutation identified	
13	339.21	213.00	760.64	7695.14	110.07	Normal	BTK	No mutation identified	
14	12.71	15.50	776.93	6535.57	94.00	BTK	BTK	*BTK* c.1714_1715delTA (p.S572Ifs14^*^)	
15	10.36	12.07	444.21	3723.29	57.43	BTK	BTK	*BTK* c.953C>T (p.S318F)	
16	8.79	13.29	745.00	6118.79	65.93	BTK	BTK	*BTK* c.11811028T>G (p.Y40D)	
17	24.29	32.36	1001.79	7820.93	109.71	BTK	BTK	*BTK* c.629insA (p.P210Tfs5^*^)	
18	7.93	167.86	609.71	5347.21	85.57	BTK	BTK	*BTK* c.1651T>A (p.Y551N)	
19	25.93	28.50	427.29	4181.00	80.07	BTK	BTK	*BTK* c.1735G>C (p.D579H)	
20	16.36	23.86	786.50	5407.93	100.57	BTK	BTK	*BTK* c.752G>A (p.W251^*^)	
21	6.93	19.00	801.07	8769.71	77.21	BTK	BTK	*BTK* c.117_119delCTA (p.del40Y)	
22	18.50	9.29	684.64	5988.29	76.57	BTK	BTK	*BTK* c.521-1G>A (splice)	
23	4.93	8.29	508.79	5970.86	77.71	BTK	BTK	*BTK* c.1876delG (p.A582Lfs4^*^)	
24	10.57	19.64	537.50	7728.86	91.00	BTK	BTK	*BTK* c.763C>T (p.R255^*^)	
25	13.50	20.36	602.29	6838.79	65.00	BTK	BTK	*BTK* c.1782delG (p.K595Rfs52^*^)	
26	9.21	20.36	1030.00	8832.43	147.07	BTK	BTK	*BTK* c.1657delA (p.S553Afs2^*^)	
27	10.36	21.71	751.29	6371.79	93.64	BTK	BTK	*BTK* c.1610delT (p.V537Dfs18^*^)	
28	14.43	8.21	557.93	5896.29	50.57	BTK	BTK	*BTK* c.37C>T (p.R13^*^)	
29	784.25	547.86	182.68	2041.46	360.21	WAS	WAS	*WAS* c.1453+2T>A	Pt. #30 (Pre-BMT)
30	1397.93	786.00	938.93	12758.86	105.64	Normal	WAS (Post-BMT)	Normal BMT donor	Pt. #29 (Post-BMT)
31	415.00	202.36	113.86	1812.00	69.21	WAS	WAS/XLT	*WAS* c.223G>A (p.V75M)	
32	768.93	492.21	11.29	375.86	82.07	WAS	WAS	*WAS* c.631C>T (p.R211^*^)	
33	342.36	346.86	11.71	248.14	77.43	WAS	WAS	*WAS* c.838C>T (p.Q280^*^)	
34	275.57	206.57	10.64	238.50	42.29	WAS	WAS	*WAS* c.838delC (p.Q280Sfs28^*^)	
35	258.79	187.50	10.29	204.64	55.43	WAS	WAS	*WAS* c.631C>T (p.R211^*^)	Brother of Pt. #36
36	448.07	236.00	4.71	237.43	46.29	WAS	WAS	*WAS* c.631C>T (p.R211^*^)	Brother of Pt. #35
37	307.79	167.00	33.14	476.79	126.14	WAS	WAS	*WAS* c.1264_1267insCCTTGCCTGCCTCT (P.G422Pfs20^*^)	
38	123.64	106.21	6.79	148.79	132.36	WAS	WAS	*WAS* c.332_336insCC (p.F113Pfs15^*^)	
39	788.79	520.50	20.71	359.36	36.00	WAS	WAS	*WAS* c.756G>A (p.W252^*^)	
40	851.71	648.71	624.43	6892.07	33.93	SCID	T-B-NK+ SCID	*RAG1* c.2159G>A (p.G720D), Homozygous	
41	1012.36	567.14	1295.93	20174.64	86.29	Normal	X-SCID - Hypomorphic	*IL2RG* c.460C>T (p.T154S)	
42	1414.36	872.43	1898.29	16707.14	17.57	SCID	T-B+NK+ SCID	Unknown-Gene panel and Exome negative	

* *Indicates the current Human Genome Variation Society nomenclature for a nonsense mutation leading to a stop codon at the protein level*.

**Table 5:** ATP7B 1056 signature peptide concentrations.

**Control sample**	**ATP7B 1056 (pmol/L)**	**Patient**	**ATP7B 1056 (pmol/L)**	**Immuno-SRM diagnosis**	**Clinical diagnosis**	**Genotype**	**Notes**
NC 1	114.29	1	113.07	Normal	X-linked CGD	*CYBB* Mutation	
NC 2	130.07	2	136.36	Normal	X-linked CGD	*CYBB* Mutation	
NC 3	110.14	3	217.07	BTK	BTK	*BTK* c.1587_1589delA (p.N530Tfs26^*^)	Brother of #4
NC 4	129.00	4	107.79	BTK	BTK	*BTK* c.1587_1589delA (p.N530Tfs26^*^)	Brother of #3
NC 5	65.36	5	183.46	BTK	BTK	*BTK* c.1940T>C (p.L647P)	
NC 6	96.93	6	30.29	BTK	BTK	*BTK* c.763C>T (p.R255^*^)	
NC 7	162.79	7	123.21	BTK	BTK	*BTK* c.1940T>C (p.L647P)	
NC 8	116.07	8	188.61	BTK	BTK	*BTK* c.1889T>A (p.M630K)	
NC 9	138.79	9	20.00	BTK	BTK	*BTK* c.1908+2delTAAGTGCTT (splice)	
NC 10	98.07	10	28.43	Normal	BTK	No mutation identified	
NC 11	115.14	11	21.57	BTK	BTK	*BTK* c.1768A>T (p.I590F)	
NC 12	127.57	12	32.21	BTK	BTK	No mutation identified	
NC 13	77.86	13	30.93	Normal	BTK	No mutation identified	
NC 14	108.57	14	19.43	BTK	BTK	*BTK* c.1714_1715delTA (p.S572Ifs14^*^)	
NC 15	162.14	15	25.21	BTK	BTK	*BTK* c.953C>T (p.S318F)	
NC 16	203.71	16	30.36	BTK	BTK	*BTK* c.11811028T>G (p.Y40D)	
NC 17	179.93	17	36.29	BTK	BTK	*BTK* c.629insA (p.P210Tfs5^*^)	
NC 18	127.36	18	33.50	BTK	BTK	*BTK* c.1651T>A (p.Y551N)	
NC 19	130.64	19	25.07	BTK	BTK	*BTK* c.1735G>C (p.D579H)	
NC 20	130.00	20	48.86	BTK	BTK	*BTK* c.752G>A (p.W251^*^)	
NC 21	101.64	21	50.71	BTK	BTK	*BTK* c.117_119delCTA (p.del40Y)	
NC 22	115.21	22	33.64	BTK	BTK	*BTK* c.521-1G>A (splice)	
NC 23	109.14	23	31.93	BTK	BTK	*BTK* c.1876delG (p.A582Lfs4^*^)	
NC 24	100.71	24	30.86	BTK	BTK	*BTK* c.763C>T (p.R255^*^)	
NC 25	134.64	25	62.71	BTK	BTK	*BTK* c.1782delG (p.K595Rfs52^*^)	
NC 26	89.07	26	46.14	BTK	BTK	*BTK* c.1657delA (p.S553Afs2^*^)	
NC 27	121.43	27	44.79	BTK	BTK	*BTK* c.1610delT (p.V537Dfs18^*^)	
NC 28	96.07	28	69.71	BTK	BTK	*BTK* c.37C>T (p.R13^*^)	
NC 29	88.36	29	90.25	WAS	WAS	*WAS* c.1453+2T>A	Same as Pt. #30 (Pre-BMT)
NC 30	118.64	30	112.57	Normal	WAS	Normal BMT donor	Same as Pt. #29 (Post-BMT)
NC 31	131.86	31	168.64	WAS	WAS/XLT	*WAS* c.223G>A (p.V75M)	
NC 32	132.64	32	108.79	WAS	WAS	*WAS* c.631C>T (p.R211^*^)	
NC 33	106.79	33	47.71	WAS	WAS	*WAS* c.838C>T (p.Q280^*^)	
NC 34	139.36	34	30.07	WAS	WAS	*WAS* c.838delC (p.Q280Sfs28^*^)	
NC 35	92.93	35	31.14	WAS	WAS	*WAS* c.631C>T (p.R211^*^)	Brother of Pt. #36
NC 36	120.29	36	60.14	WAS	WAS	*WAS* c.631C>T (p.R211^*^)	Brother of Pt. #35
NC 37	85.86	37	55.79	WAS	WAS	*WAS* c.1264_1267insCCTTGCCTGCCTCT (P.G422Pfs20^*^)	
NC 38	124.00	38	7.21	WAS	WAS	*WAS* c.332_336insCC (p.F113Pfs15^*^)	
NC 39	99.50	39	63.36	WAS	WAS	*WAS* c.756G>A (p.W252^*^)	
NC 40	117.43	40	139.50	SCID	T-B-NK+ SCID	*RAG1* c.2159G>A (p.G720D) - Homozygous	
		41	182.79	Normal	X-SCID-Hypomorphic	*IL2RG* c.460C>T (p.T154S)	
		42	176.79	SCID	T-B+NK+ SCID	Unknown-Gene panel and Exome negative	

* *Indicates the current Human Genome Variation Society nomenclature for a nonsense mutation leading to a stop codon at the protein level*.

Peptide concentration cutoffs for each PIDD diagnosis were arbitrarily set at −2 SD (BTK 545), −2.25 SD (BTK 407), −2.15 SD (WASp 274), −1.75 SD (WASp 289), and −1.25 SD (CD3ϵ). This translated to cutoff concentrations of 106.90 pmol/L (BTK 545), 49.19 pmol/L (BTK 407), 195.10 pmol/L (WASp 274), 2428.99 pmol/L (WASp 289), and 39.96 pmol/L (CD3ϵ). Use of these ranges resulted in 2 false positives in the normal controls. NC4 and NC20 were indicated to be WAS and SCID patients, respectively. NC signature peptide values are shown in Table 6.

**Table 6:** Quantification of signature peptides in normal controls from a blinded cohort study.

**Sample**	**BTK 545 (pmol/L)**	**BTK 407 (pmol/L)**	**WASp 274 (pmol/L)**	**WASp 289 (pmol/L)**	**CD3ϵ 197 (pmol/L)**	**Sample**	**BTK 545 ATP7B Ratio**	**BTK 407ATP7B Ratio**	**WASp 274 ATP7B Ratio**	**WASp 289ATP7B Ratio**	**CD3ϵ 197 ATP7B ratio**
NC 1	251.07	252.07	699.43	5245.29	175.14	NC 1	2.20	2.21	6.12	45.90	1.53
NC 2	1485.71	796.57	1504.79	11111.50	149.43	NC 2	11.42	6.12	11.57	85.43	1.15
NC 3	926.79	679.86	966.21	6995.86	242.07	NC 3	8.41	6.17	8.77	63.52	2.20
NC 4	177.93	169.71	145.93	1674.93	51.29	NC 4	1.38	1.32	1.13	12.98	0.40
NC 5	923.00	597.29	1076.21	12230.57	259.71	NC 5	14.12	9.14	16.47	187.13	3.97
NC 6	1322.79	690.50	1445.07	14981.07	427.07	NC 6	13.65	7.12	14.91	154.56	4.41
NC 7	1700.64	1253.00	1928.43	9975.57	104.07	NC 7	10.45	7.70	11.85	61.28	0.64
NC 8	609.36	299.57	684.50	6569.93	84.86	NC 8	5.25	2.58	5.90	56.60	0.73
NC 9	927.43	642.14	1029.29	10630.00	272.07	NC 9	6.68	4.63	7.42	76.59	1.96
NC 10	1145.71	599.36	1068.93	12893.64	392.79	NC 10	11.68	6.11	10.90	131.47	4.01
NC 11	970.29	690.50	886.07	9373.64	536.00	NC 11	8.43	6.00	7.70	81.41	4.66
NC 12	916.93	673.79	927.79	12554.64	413.71	NC 12	7.19	5.28	7.27	98.41	3.24
NC 13	1071.86	582.71	1817.00	15947.21	82.71	NC 13	13.77	7.48	23.34	204.83	1.06
NC 14	834.86	429.21	1861.93	18301.64	57.07	NC 14	7.69	3.95	17.15	168.57	0.53
NC 15	1527.86	705.07	1499.36	11235.71	148.00	NC 15	9.42	4.35	9.25	69.30	0.91
NC 16	932.64	677.64	1171.71	14357.29	251.07	NC 16	4.58	3.33	5.75	70.48	1.23
NC 17	1667.21	764.93	1634.29	19107.36	98.07	NC 17	9.27	4.25	9.08	106.19	0.55
NC 18	601.71	317.00	814.00	8712.79	162.71	NC 18	4.72	2.49	6.39	68.41	1.28
NC 19	2520.93	1336.43	2293.86	21659.43	104.14	NC 19	19.30	10.23	17.56	165.79	0.80
NC 20	967.36	644.50	1059.14	7774.50	27.64	NC 20	7.44	4.96	8.15	59.80	0.21
NC 21	984.00	606.50	1111.43	6288.00	254.43	NC 21	9.68	5.97	10.93	61.86	2.50
NC 22	981.14	549.00	1043.43	13103.14	526.86	NC 22	8.52	4.77	9.06	113.73	4.57
NC 23	1398.86	770.71	1341.07	12621.43	439.79	NC 23	12.82	7.06	12.29	115.64	4.03
NC 24	489.71	319.57	674.57	4735.50	138.29	NC 24	4.86	3.17	6.70	47.02	1.37
NC 25	892.29	541.00	867.29	7971.86	296.29	NC 25	6.63	4.02	6.44	59.21	2.20
NC 26	1620.93	911.79	1682.29	16762.21	263.57	NC 26	18.20	10.24	18.89	188.19	2.96
NC 27	317.93	251.50	248.21	3036.50	100.43	NC 27	2.62	2.07	2.04	25.01	0.83
NC 28	701.71	422.14	1588.29	15102.14	42.29	NC 28	7.30	4.39	16.53	157.20	0.44
NC 29	1562.21	876.14	1386.14	10278.71	639.79	NC 29	17.68	9.92	15.69	116.33	7.24
NC 30	935.43	483.57	1007.29	10191.71	249.21	NC 30	7.88	4.08	8.49	85.90	2.10
NC 31	461.21	336.86	639.00	4190.21	191.57	NC 31	3.50	2.55	4.85	31.78	1.45
NC 32	835.93	671.86	780.14	5269.57	243.14	NC 32	6.30	5.07	5.88	39.73	1.83
NC 33	1248.43	759.00	1419.71	11329.57	273.50	NC 33	11.69	7.11	13.29	106.10	2.56
NC 34	474.43	314.86	563.57	3114.79	171.14	NC 34	3.40	2.26	4.04	22.35	1.23
NC 35	1648.64	1088.07	1730.00	9638.43	238.57	NC 35	17.74	11.71	18.62	103.72	2.57
NC 36	959.50	773.43	1002.36	9043.00	170.21	NC 36	7.98	6.43	8.33	75.18	1.42
NC 37	1440.57	1026.86	1427.57	7468.00	61.79	NC 37	16.78	11.96	16.63	86.98	0.72
NC 38	948.21	620.29	1135.29	8484.86	299.50	NC 38	7.65	5.00	9.16	68.43	2.42
NC 39	717.21	435.71	1610.86	13417.57	43.07	NC 39	7.21	4.38	16.19	134.85	0.43
NC 40	1437.36	843.00	1306.07	9699.21	464.00	NC 40	12.24	7.18	11.12	82.60	3.95

**Figure 6:**
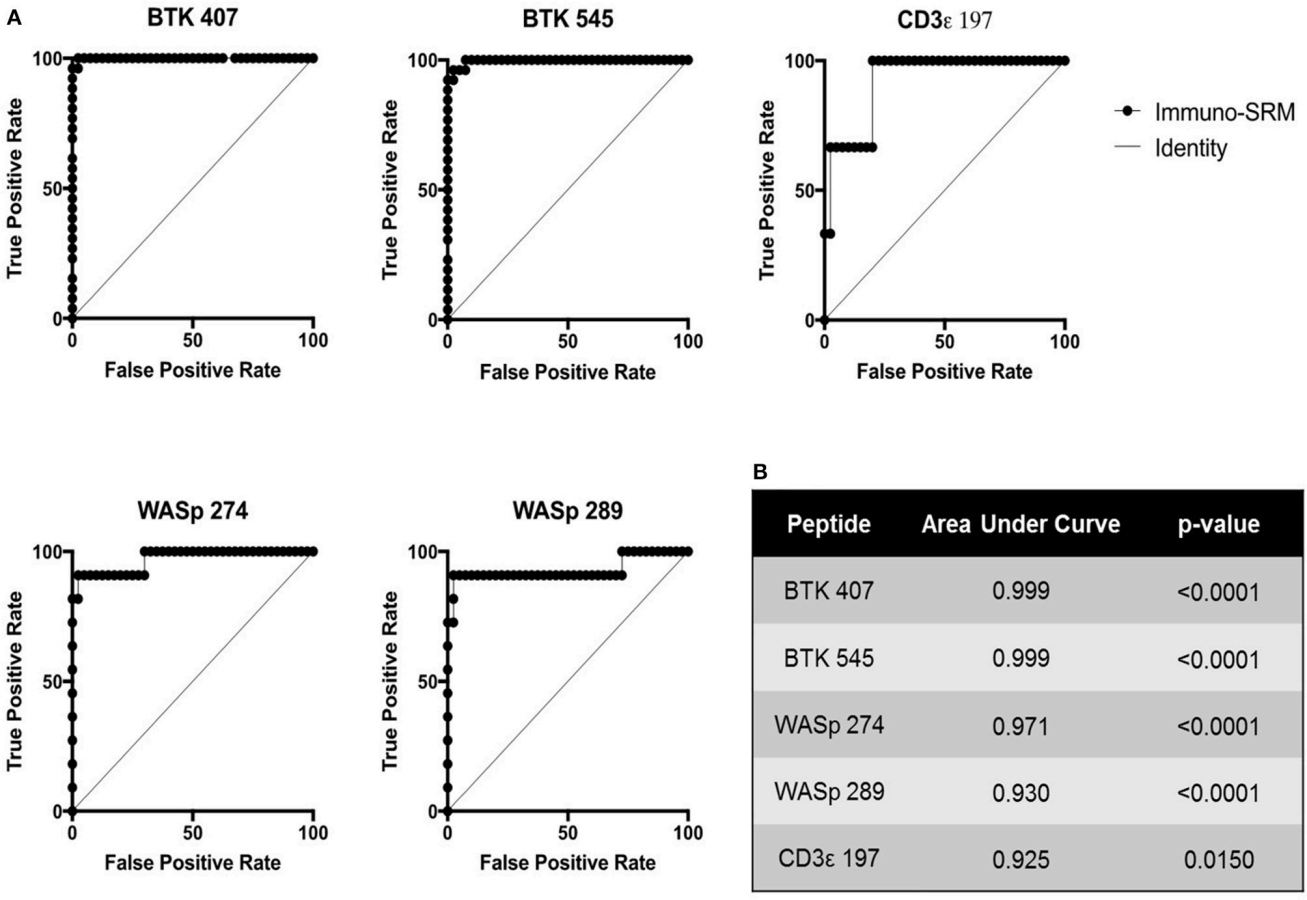
ROC plots showing the diagnostic performance of Immuno-SRM for PIDDs. **(A)** ROC plots for each signature peptide. True positive and false positive rates are plotted for increasingly stringent cutoff values. Line of identity indicates a test that cannot distinguish patients from controls. **(B)** Area under the curve (AUC) values and *p*-values for each peptide of interest.

Using these cutoffs, the specific PIDD diagnosis was predicted for each patient. Predicted diagnoses showed excellent agreement with clinical or genetic diagnoses as shown in Table 4. Every molecularly-confirmed case of WAS and BTK was also diagnosed by immuno-SRM analysis. Two patients, Patient 10 and 13, who were clinically diagnosed as agammaglobulinemia, had normal levels of BTK protein by immuno-SRM. Molecularly, no variants in *BTK* were identified in these patients (34). Interestingly, patient 12 with agammaglobulinemia had low levels of BTK protein, but no variants were found in the coding regions of *BTK*. In addition, one case of X-linked hypomorphic SCID, patient 41, was identified as normal by immuno-SRM. For each signature peptide utilized, area under the curve (AUC) analysis of the ROC plots reveal areas from 0.925 to 0.999 with *p*-values ranging from 0.015–0.0001 (Figure 6). Overall, 97.6% of cases had concordance between the clinical diagnosis and immuno-SRM assay results. Interesting outlier and discordant cases are described further in the discussion.

Signature peptide concentrations for randomly selected NBS lab samples are shown in Table 7. Each DBS sample had measured peptide concentrations above the previously set diagnostic cutoffs for PIDDs, indicating unaffected status.

**Table 7:** Signature peptides levels in DBS obtained from Washington State Newborn Screening Laboratory (Samples collected prior to March 2015).

**Sample**	**BTK 545 (pmol/L)**	**BTK 407(pmol/L)**	**WASp 274 (pmol/L)**	**WASp 289(pmol/L)**	**CD3ϵ (pmol/L)**	**Sample**	**BTK 545 (pmol/L)**	**BTK 407(pmol/L)**	**WASp 274 (pmol/L)**	**WASp 289(pmol/L)**	**CD3ϵ pmol/L)**
NBS 1	355.71	623.28	1579.24	31988.24	82.85	NBS 32	224.50	426.40	1009.47	20974.93	76.84
NBS 2	358.71	649.43	1587.71	16737.14	55.14	NBS 33	138.51	257.70	668.07	12568.65	65.82
NBS 3	242.96	424.54	856.51	17722.60	98.01	NBS 34	219.06	440.13	1221.81	24626.48	85.14
NBS 4	301.43	522.86	928.86	11055.71	64.00	NBS 35	263.99	482.34	1316.96	27654.18	89.00
NBS 5	209.14	366.14	703.14	8332.86	49.43	NBS 36	213.20	397.64	763.65	15036.88	69.54
NBS 6	123.86	258.57	601.43	7441.43	66.29	NBS 37	120.76	205.76	447.29	3409.73	51.22
NBS 7	196.74	369.88	900.01	6677.81	56.52	NBS 38	108.89	199.60	433.98	9057.33	66.82
NBS 8	265.86	486.00	740.00	9151.43	71.86	NBS 39	107.60	201.18	566.91	11997.74	62.53
NBS 9	165.41	345.98	696.68	5404.35	76.69	NBS 40	220.50	379.03	758.07	15722.26	69.40
NBS 10	231.29	430.14	880.86	10188.57	88.14	NBS 41	161.69	310.50	671.93	13942.27	70.11
NBS 11	207.33	352.99	732.31	14463.10	73.40	NBS 42	249.11	390.48	999.88	6563.34	61.10
NBS 12	186.14	316.29	690.00	8271.43	78.14	NBS 43	264.57	505.38	1119.36	23852.39	95.01
NBS 13	260.42	606.97	1117.93	19750.12	90.14	NBS 44	759.79	1255.58	2862.29	51995.92	137.65
NBS 14	252.55	452.72	932.35	18150.42	86.42	NBS 45	120.48	222.93	432.41	9284.83	78.98
NBS 15	203.61	393.91	940.65	17818.46	99.30	NBS 46	816.16	1234.69	2550.93	19173.49	255.98
NBS 16	261.13	398.35	668.64	4955.06	50.94	NBS 47	823.46	1307.23	3021.83	22767.80	250.97
NBS 17	519.83	903.73	933.06	5646.16	71.54	NBS 48	838.91	1241.70	2014.65	15955.49	203.18
NBS 18	456.87	553.17	963.40	6194.18	71.40	NBS 49	623.42	905.88	1295.50	9367.82	62.67
NBS 19	641.17	1019.63	1579.24	10760.05	100.45	NBS 50	789.83	1219.09	2700.03	19750.12	112.32
NBS 20	167.55	334.39	607.69	4773.34	52.23	NBS 51	454.58	736.89	1234.69	8237.44	65.53
NBS 21	329.81	614.70	1072.14	7413.27	67.54	NBS 52	624.14	810.87	805.00	6364.45	54.94
NBS 22	427.25	656.19	1086.45	7254.45	75.84	NBS 53	360.58	498.08	785.40	5390.04	58.81
NBS 23	325.95	447.72	587.37	3195.10	93.29	NBS 54	166.55	300.91	712.42	4889.24	64.82
NBS 24	233.52	385.19	486.06	3554.25	73.83	NBS 55	141.23	267.43	811.15	5523.11	72.97
NBS 25	255.69	381.47	703.27	5066.67	92.72	NBS 56	263.71	438.99	975.13	6845.22	108.46
NBS 26	246.39	365.30	937.78	5945.21	86.42	NBS 57	163.12	290.75	767.37	5191.15	67.11
NBS 27	185.30	291.04	786.97	6131.22	104.74	NBS 58	253.55	448.86	1016.05	6823.76	62.53
NBS 28	208.91	387.19	755.35	5421.52	116.33	NBS 59	267.28	507.53	1207.50	7793.88	71.69
NBS 29	253.98	358.43	865.81	6672.09	129.92	NBS 60	149.10	269.29	698.54	4989.40	63.96
NBS 30	168.27	289.03	565.05	4417.06	111.03	NBS 61	118.33	240.81	555.17	3923.41	71.54
NBS 31	139.37	274.44	580.07	12012.05	52.37	NBS 62	244.68	288.03	326.81	2493.98	72.12
						Mean	300.86	499.05	997.04	11366.65	85.25
						SD	191.74	281.62	563.07	8466.44	39.49

## Discussion

We have demonstrated immuno-SRM as a sensitive and specific proteomic screening method for the multiplex detection of patients with three life-threatening PIDD (i.e., SCID, WAS, and XLA) from DBS. Our results clearly differentiate patients with PIDD from normal controls, with low levels of endogenous peptides of transmembrane protein CD3ϵ and intracellular proteins WASp and BTK correlating with the target diseases (SCID, WAS, and XLA, respectively). These diagnoses can be made in a single run with a total runtime of 20 min or ~6.67 min per disease target. Our results also demonstrate peptide stability in DBS, with minimal variability in concentrations after 72 h of storage at room temperature (Table 3).

The immuno-SRM platform reliably detected endogenous peptide from normal control DBS in this highly multiplexed fashion. Normal control DBS (*N* = 40) were unblinded and utilized to define the normal ranges and potential screen-positive cutoffs (Table 6). In clinical laboratories, reference ranges for diagnostic tests are determined by the normal distribution in the general population. Given that these samples were obtained under strict standard operating procedures, variability amongst this group is likely due to inherent inter-patient variability. These samples were not controlled for age, gender, ethnicity, or differences in blood characteristics and likely represent a broad snapshot of the population. These possible differences in patient subgroups need to be further explored in larger pilot studies. Initial cutoffs for screening tests are typically conservative, aiming to detect all true positives without creating an excessively high screen positive rate relative to the incidence of disease. However, these cutoffs are continually validated and adjusted in accordance with population-based studies. Given these parameters, the definition of screen-positive results ranged from 1.25 to 2.25 SD below the mean for the peptides in this study. The chosen cutoffs generated 2 false positive normal controls, one WAS (NC4) and one SCID (NC20) (Table 6). In the case of NC4, rescreening showed WASp levels in the normal range. These preliminary cutoffs are not static and will become better defined as higher numbers of normal controls and patient samples are screened.

Using these cutoffs, we were able to positively identify every molecularly-confirmed BTK and WAS patient covering a broad range of variants (Table 4). As hypothesized, peptide concentrations are reduced in the majority of BTK and WAS cases independent of genotype (18, 19, 40). These peptide levels are therefore promising biomarkers for diagnosis and screening. Of the 3 SCID patients available for testing, 2 were positively identified by CD3ϵ analysis. The third patient, while having low CD3ϵ levels relative to the majority of the normal controls, was within the defined cutoffs and was found to have a “hypomorphic” variant in IL2RG known to generate a partially functional protein. This is reflected by the patients total CD3+ T-cell count that was mildly low (800 cells/μL) but not absent as in the classical form of SCID. Since CD3ϵ is exclusively expressed by CD3+ T cells in peripheral blood, the amount of CD3ϵ protein present is reflective of total CD3+ T cell counts. Therefore, patients with hypomorphic forms of SCID, “leaky” forms of SCID who have expanded oligoclonal T cell populations, or expanded maternally-derived T cells may be missed by the Immuno-SRM approach (1). Given the genetic heterogeneity of SCID, it may not be possible to universally screen all subtypes with a single universal marker. Second generation immuno-SRM screens will benefit from the inclusion of more specific protein biomarkers for the more common subtypes, such as those featuring ADA, recombination activating gene 1 (RAG1), or interleukin 2 receptor subunity gamma (ILR2G) deficiency.

ROC curves were constructed to assess the diagnostic ability of immuno-SRM analysis. These plots relate the true positive rate to the false positive rate with increasingly stringent cutoff values. As diagnostic cutoffs are lowered, the test will have greater ability to note true positives, but this process is also more likely to lead to false positives. A screening test maintaining a high true positive rate and a low false positive rate will therefore lead to graphs lying close to the y axis and a large AUC (Figure 6). These values for immuno-SRM indicate high diagnostic potential for immuno-SRM analysis of signature peptides of PIDDs.

QC monitoring of digestion and process performance is included in the current immuno-SRM multiplex in the form of ATP7B signature peptide measurements. As not all detected metabolites are helpful NBS targets, the calculation of metabolite ratios and secondary metabolite analysis are employed to improve the sensitivity and specificity of NBS for certain diseases, such as C3:C2 ratio and 2-methylcitric acid analysis in methylmalonic aciduria (41). In addition, target ratioing can account for variability between samples brought on by a number of factors including sample collection quality, storage, extraction and digestion efficiency, and blood characteristics (30). Here, ATP7B concentrations were found to be largely consistent across the screened samples (Table 5). In the future, absent ATP7B could serve to flag improperly processed or handled specimens. As a pilot, we compared each PIDD peptide by ratio to the endogenous concentration of ATP7B in the same sample. The resulting predictions based on peptide concentrations showed complete agreement with the clinical diagnosis, demonstrating immuno-SRM, and ATP7B ratioing can be an effective and complementary tool for PIDD diagnosis (Tables 8, 9). These types of ratios have potential utility in clinical immuno-SRM screening, provided the chosen peptide is proven to be a ubiquitous and invariant signal across a large cohort of samples.

**Table 8:** Ratios of signature peptides against ATP7B peptide and patient diagnosis in a blinded cohort study.

**Patient**	**BTK 545 ATP7B Ratio**	**BTK 407ATP7B ratio**	**WASp 274 ATP7B ratio**	**WASp 289ATP7B ratio**	**CD3ϵ 197 ATP7B ratio**	**Immuno-SRM diagnosis**	**Clinical diagnosis**	**Genotype**	**Notes**
1	19.65	12.05	23.08	245.54	2.18	Normal	X-linked CGD	CYBB Mutation	
2	8.43	5.46	7.54	87.13	1.69	Normal	X-linked CGD	CYBB Mutation	
3	0.02	0.06	8.84	110.07	0.40	BTK	BTK	BTK c.1587_1589delA (p.N530Tfs26^*^)	Brother of #4
4	0.11	0.13	15.06	166.51	1.86	BTK	BTK	BTK c.1587_1589delA (p.N530Tfs26^*^)	Brother of #3
5	0.15	0.09	6.98	94.91	0.54	BTK	BTK	BTK c.1940T>C (p.L647P)	
6	0.67	0.38	8.49	142.32	5.86	BTK	BTK	BTK c.763C>T (p.R255^*^)	
7	0.16	0.10	9.10	147.53	0.58	BTK	BTK	BTK c.1940T>C (p.L647P)	
8	0.12	0.13	3.38	46.12	0.55	BTK	BTK	BTK c.1889T>A (p.M630K)	
9	1.09	0.67	24.75	300.48	3.18	BTK	BTK	BTK c.1908+2delTAAGTGCTT	
10	8.36	3.98	19.28	251.94	2.31	Normal	BTK	No mutation identified	
11	0.50	0.51	36.24	444.99	3.79	BTK	BTK	BTK c.1768A>T (p.I590F)	
12	0.43	0.35	19.20	218.05	2.04	BTK	BTK	No mutation identified	
13	10.97	6.89	24.59	248.80	3.56	Normal	BTK	No mutation identified	
14	0.65	0.80	39.99	336.39	4.84	BTK	BTK	BTK c.1714_1715delTA (p.S572Ifs14^*^)	
15	0.41	0.48	17.62	147.67	2.28	BTK	BTK	BTK c.953C>T (p.S318F)	
16	0.29	0.44	24.54	201.56	2.17	BTK	BTK	BTK c.11811028T>G (p.Y40D)	
17	0.67	0.89	27.61	215.54	3.02	BTK	BTK	BTK c.629insA (p.P210Tfs5^*^)	
18	0.24	5.01	18.20	159.62	2.55	BTK	BTK	BTK c.1651T>A (p.Y551N)	
19	1.03	1.14	17.04	166.76	3.19	BTK	BTK	BTK c.1735G>C (p.D579H)	
20	0.33	0.49	16.10	110.69	2.06	BTK	BTK	BTK c.752G>A (p.W251^*^)	
21	0.14	0.37	15.80	172.92	1.52	BTK	BTK	BTK c.117_119delCTA (p.del40Y)	
22	0.55	0.28	20.35	178.00	2.28	BTK	BTK	BTK c.521-1G>A (splice)	
23	0.15	0.26	15.94	187.01	2.43	BTK	BTK	BTK c.1876delG (p.A582Lfs4^*^)	
24	0.34	0.64	17.42	250.47	2.95	BTK	BTK	BTK c.763C>T (p.R255^*^)	
25	0.22	0.32	9.60	109.05	1.04	BTK	BTK	BTK c.1782delG (p.K595Rfs52^*^)	
26	0.20	0.44	22.32	191.41	3.19	BTK	BTK	BTK c.1657delA (p.S553Afs2^*^)	
27	0.23	0.48	16.78	142.27	2.09	BTK	BTK	BTK c.1610delT (p.V537Dfs18^*^)	
28	0.21	0.12	8.00	84.58	0.73	BTK	BTK	BTK c.37C>T (p.R13^*^)	
29	8.69	6.07	2.02	22.62	3.99	WAS	WAS	WAS c.1453+2T>A	Same as Pt. #30 (Pre-BMT)
30	12.42	6.98	8.34	113.34	0.94	Normal	WAS (Post-BMT)	Normal BMT donor	Same as Pt. #29 (Post-BMT)
31	2.46	1.20	0.68	10.74	0.41	WAS	WAS/XLT	WAS c.223G>A (p.V75M)	
32	7.07	4.52	0.10	3.46	0.75	WAS	WAS	WAS c.631C>T (p.R211^*^)	
33	7.18	7.27	0.25	5.20	1.62	WAS	WAS	WAS c.838C>T (p.Q280^*^)	
34	9.16	6.87	0.35	7.93	1.41	WAS	WAS	WAS c.838delC (p.Q280Sfs28^*^)	
35	8.31	6.02	0.33	6.57	1.78	WAS	WAS	WAS c.631C>T (p.R211^*^)	Brother of Pt. #36
36	7.45	3.92	0.08	3.95	0.77	WAS	WAS	WAS c.631C>T (p.R211^*^)	Brother of Pt. #35
37	5.52	2.99	0.59	8.55	2.26	WAS	WAS	WAS c.1264_1267insCCTTGCCTGCCTCT (P.G422Pfs20^*^)	
38	17.14	14.72	0.94	20.62	18.35	WAS	WAS	WAS c.332_336insCC (p.F113Pfs15^*^)	
39	12.45	8.22	0.33	5.67	0.57	WAS	WAS	WAS c.756G>A (p.W252^*^)	
40	6.11	4.65	4.48	49.41	0.24	SCID	T-B-NK+ SCID	RAG1 c.2159G>A (p.G720D), Homozygous	
41	5.54	3.10	7.09	110.37	0.47	Normal	X-SCID—Hypomorphic	IL2RG c.460C>T (p.T154S)	
42	8.00	4.93	10.74	94.51	0.10	SCID	T-B+NK+ SCID	Unknown—Gene panel and exome negative	

* *Indicates the current Human Genome Variation Society nomenclature for a nonsense mutation leading to a stop codon at the protein level*.

**Table 9:** Cutoffs for signature peptides by the ratios against ATP7B peptide.

	**BTK 545 ATP7B ratio**	**BTK 407 ATP7B**	**WASp 274 ATP7B ratio**	**WASp 289 ATP7B**	**CD3ϵ 197 ATP7B ratio**
Average	9.39	5.73	10.79	94.29	2.10
SD	4.44	2.59	4.87	46.60	1.54
Cutoff	1.23	1.16	2.04	24.16	0.33

One case demonstrated the benefit of having both primary and secondary signature peptides for each protein of interest. Patient 18 was predicted to have XLA using analysis of BTK 545 instead of primary marker BTK 407. Levels of BTK 407 were significantly reduced relative to the average, 167.86 vs. 642.16 pmol/L, but not quite low enough to trigger a positive screen. In contrast, BTK 545 levels were nearly absent (Table 4) because the patient harbors the p.Y551N variant, which is located within the amino acid sequence 545–558 encompassed by the signature peptide itself. In this case, our multiplexed peptides allowed for confirmation of a positive diagnosis that was initially borderline.

It was notable that we found normal levels of BTK in two clinically defined agammaglobulinemic individuals (patients 10 and 13) who lacked variants in *BTK* by Sanger sequencing (Table 4). These patients therefore likely do not have XLA but may have autosomal forms of agammaglobulinemia, although broader genetic testing was not performed. Patient 12 had diminished levels of BTK protein but no identifiable variant in *BTK*. This suggests the variant may have been missed during sequencing of the coding region and intron-exon junctions because the patient's *BTK* variant may affect either the regulatory elements, Poly-adenylation signal, or intronic regions. These cases highlight the clinical utility of immuno-SRM.

Additionally, two samples obtained from the same WAS patient pre- and post-bone marrow transplant (BMT) were analyzed (samples #29 and 30 in Table 4, respectively). Pre-BMT, immuno-SRM analysis identified the patient as having WAS. Post-BMT, the patient was identified as normal. This case highlights the ability of immuno-SRM to follow the therapeutic course of BMT and confirm successful reconstitution of the immune system. It is possible that a similar principle would apply to patients with monogenetic disorders undergoing gene therapy.

Overall, the analysis showed the immuno-SRM assay to have a broad linear range and acceptable precision in determining the concentrations of target peptides in DBS (Table 3). Correlation plots show significant concordance of sample analysis by different MS instruments in two separate laboratory facilities (Figures 3, 4). Four of the five primary peptides, BTK 407, WASp 274, ATP7B 1056, and CD3ϵ 197 were nearly identical upon analysis with *R*^2^ values > 0.97. WASp 289 showed slightly more variable performance with an overlap of *R*^2^ = 0.85 and would therefore likely be a secondary marker to WASp 274 when conducting clinical analysis. Additionally, BTK 545 showed a variability greater than 20% CV, which would make it suitable as a secondary marker to BTK 407. These results show that immuno-SRM analysis has high potential for clinical application and transferability. Further work is underway to validate the inter-laboratory transferability of the assay.

Randomly selected samples provided by the NBS laboratory of Washington State were used to test the feasibility of utilizing immuno-SRM analysis in the context of NBS. Due to limited sample availability and to test the ability of immuno-SRM to analyze signature peptides from a smaller sample, the amount of DBS used was reduced from 1 whole spot to Five or Six 3-mm punches. Peptides of interest were enriched and analyzed with minimal change to sample processing. The concentrations of signature peptides were all still greater than the pre-defined cutoffs obtained from analysis of known normal controls (Table 7). These newborns would therefore be designated as normal. Upon analysis it was evident that, while average concentrations of WASp related peptides were in agreement with previously generated normal control values, BTK and CD3ϵ concentrations were significantly reduced. As these samples were obtained in 2014, it is unclear whether this reduction is evidence of a biological difference between newborns and adults or secondary to preferential degradation of these proteins relative to WASp. The ability to robustly perform this analysis with a greatly reduced sample input makes immuno-SRM analysis more amenable to translation into NBS. With optimization, we are confident the assay can be improved with further reduction in sample consumption, higher efficiency in multiplexing, decreased analytical runtimes, automation, and higher affinity monoclonal antibodies. The high specificity of mass spectrometry makes increasing the number of multiplexed targets straightforward, including the addition of peptides as secondary markers or expansion to include other conditions. This high-throughput multiplexed method may effectively decrease run time per disease, making it suitable for NBS where current automated methods have a typical run time of less than 3 min (42). The successful prediction of BTK patients using DBS shipped at ambient temperature via traditional post from Vietnam also highlights the potential utility for diagnostic testing in resource poor settings where collection and shipping of DBS has to be economical.

A number of limitations exist in this current generation immuno-SRM screen that can be improved upon to better define the assay for use in a population-based screen. In terms of process, current assays feature longer runtimes and greater sample consumption than would be ideal for translation into the newborn screening laboratory. Work is ongoing to optimize LC-MS/MS gradients, column types, and extraction procedures to be able to maximize peptide signal and reliability in the shortest possible assay. Further sample reduction will be possible with the development of higher signal signature peptide targets such that lower concentrations will give greater or equivalent MS response. In addition, optimization of peptide elution procedures would provide reduced background to allow for both greater signal and faster screening times. A greater reliability and confidence will come with incorporation of additional peptide sequences for proteins of interest. Currently, for instance, there is available only one target sequence for CD3ϵ. Additional secondary markers will provide a more robust assay. Finally, the limited number of samples analyzed here allow for the definition of only tentative normal control and disease ranges. A greatly expanded pilot study will provide better defined ranges and inclusion of a broad range of patient backgrounds will delineate possible inherent differences due to age, gender, or ethnicity.

NBS has been one of the most successful public health initiatives in modern times, but traditionally relies on the detection of accumulated metabolites due to downstream enzyme deficiency. However, many genetic disorders including PIDD are characterized by absent or decreased proteins, limiting the scope of current NBS methods (18, 19). By being able to detect PIDD-related peptides from DBS, immuno-SRM may bridge this gap in current coverage, allowing for the expansion of NBS to treatable diseases currently without metabolite biomarkers. Immuno-SRM would rapidly provide quantified evidence of protein deficiency and could be performed simultaneously with initial screening and molecular analysis from DBS without further invasive procedures. In fact, complementary testing with both targeted proteomic analysis and molecular testing would provide significant value by not only rapidly identifying potential patients but also providing information on the effects of variants of unknown significance where they are found. Quantification of these signature peptides lays the foundation for immuno-SRM as a highly multiplexable screening and diagnostic tool for various congenital diseases.

## Author Contributions

SH, CC, JW, and AP conceived the study. CC, SJ, and RD performed experimentation and data analysis. GS, TT, and HO provided patient samples and analyzed the data. IC wrote the first draft of the manuscript, and CC contributed significantly to the methods and discussion. All authors reviewed and revised the manuscript, and approved the final version for submission.

### Conflict of Interest Statement

AP is founder of Precision Assays. The remaining authors declare that the research was conducted in the absence of any commercial or financial relationships that could be construed as a potential conflict of interest.

## Abbreviations

ACD: Acid Citrate Dextrose
ADA: Adenosine Deaminase
ATP7B: ATPase copper transporting protein
BTK: Bruton's Tyrosine Kinase
CD3: Cluster of Differentiation 3
DTT: Dithiothreiol
FA: Formic Acid
HLA: Human Leukocyte Antigen
IS: internal standard
ILR2G: Interleukin 2 receptor subunity gamma
LC-MS/MS: Liquid Chromatography Tandem Mass Spectrometry
SRM: Selected Reaction Monitoring
Immuno-SRM: Immunoaffinity enrichment coupled to selected reaction monitoring
SISCAPA: Stable Isotope Standards and Capture by Anti-Peptide Antibodies
PBMC: Peripheral Blood Mononuclear Cells
PIDD: Primary Immunodeficiency Disorders
SCID: Severe Combined Immunodeficiency
SNP: Single Nucleotide Polymorphism
UPLC: Ultra Performance Liquid Chromatography
WAS: Wiskott-Aldrich Syndrome
WASp: Wiskott-Aldrich Syndrome Protein
WBC: White Blood Cell
XLA: X-Linked Agammaglobulinemia
TREC: T Cell Receptor Excision Circle
KREC: Kappa-deleting Element recombination Circle
AUC: Area Under the Curve
RAG1: Recombination activating gene 1
ROC: Reciever Operating Characteristic
CV: Coefficient of Variation
LLOQ: Lower Limit of Quantification.
